# Supplementary material for: A whole-animal phenotypic drug screen identifies suppressors of atherogenic lipoproteins
Source: eLife. 2026 Aug 27;14:RP105314. doi: 10.7554/eLife.105314 (PMC13521566; doi:10.7554/eLife.105314)
Supplement: Supplementary file 2 [file elife-105314-code1.zip › JHDL_primary_result/PIPERACILLIN SODIUM/PIPERACILLIN SODIUM_JHDL_5_004_004_2019_05_17_10_59.pdf]

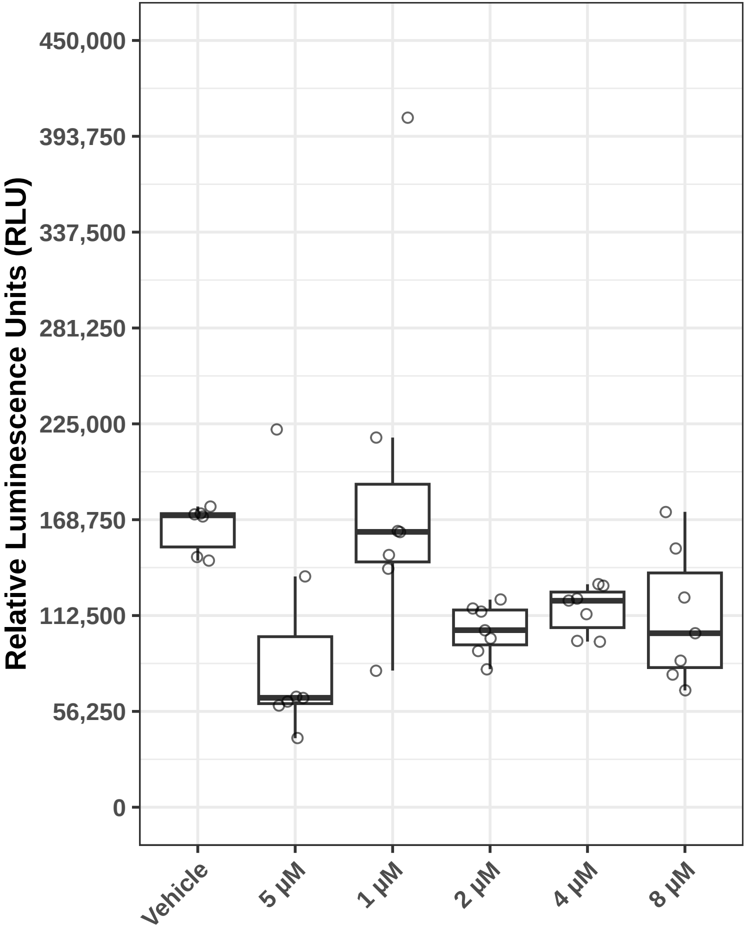

Relative Luminescence Units (RLU)
450,000
393,750
337,500
281,250
225,000
168,750
112,500
56,250
0
Vehicle
5 μM
1 μM
2 μM
4 μM
8 μM
